# Short versus long duration of dual antiplatelet therapy following drug-eluting stents: a meta-analysis of randomised trials

**DOI:** 10.1007/s12471-018-1104-6

**Published:** 2018-03-14

**Authors:** R. Rozemeijer, M. Voskuil, J. P. Greving, M. L. Bots, P. A. Doevendans, P. R. Stella

**Affiliations:** 1 Department of Cardiology, University Medical Centre Utrecht, Utrecht, The Netherlands; 2 Julius Centrum, Department of Epidemiology, University Medical Centre Utrecht, Utrecht, The Netherlands; 3 Netherlands Heart Institute, Utrecht, The Netherlands

**Keywords:** Coronary artery disease, Drug-eluting stent, Stent thrombosis, Major bleeding events

## Abstract

**Background:**

Dual antiplatelet therapy (DAPT) remains the cornerstone therapy in the prevention of ischaemic events following drug-eluting stent (DES) implantation. Mandatory duration of DAPT after DES however, is a matter of debate. We aimed to evaluate safety and efficacy of short-term (up to 6 months) versus long-term (12 months) DAPT after DES implantation.

**Methods:**

We searched PubMed, EMBASE, Cochrane databases, and international meetings for randomised clinical trials (RCTs) comparing short with long DAPT. We performed a systematic review and meta-analysis of major trials with primary outcomes: all-cause death, myocardial infarction, definite or probable stent thrombosis, stroke, and major bleeding event.

**Results:**

Nine RCTs with a total number of 19,099 patients were pooled in the present meta-analysis. When compared with long DAPT, short DAPT was associated with a significant reduction in major bleeding events (0.62% vs. 1.10%, risk ratio (RR) 0.58, 95% confidence interval (CI) 0.39 to 0.86, *p* < 0.007, I^2^ = 21%), whereas all-cause death (1.65% vs. 1.84%, RR 0.90, 95% CI 0.73 to 1.11, *p* = 0.34, I2 = 0%), myocardial infarction (1.91% vs. 1.68%, RR 1.14, 95% CI 0.92 to 1.40, *p* = 0.23, I2 = 0%), definite or probable stent thrombosis (0.62% vs. 0.47%, RR 1.25, 95% CI 0.84 to 1.86, *p* = 0.27, I2 = 0%), and stroke (0.60% vs. 0.67%, RR 0.91, 95% CI 0.63 to 1.31, *p* = 0.61, I2 = 0%) were similar.

**Conclusions:**

Short DAPT following DES implantation results in a significant reduction of major bleeding events with no apparent increase in all-cause death, myocardial infarction, stent thrombosis, or stroke. Future dedicated trials should investigate the optimal strategies for patient-tailored DAPT in various subgroups.

**Electronic supplementary material:**

The online version of this article (10.1007/s12471-018-1104-6) contains supplementary material, which is available to authorized users.

## Introduction

Dual antiplatelet therapy (DAPT) with aspirin and a P2Y12 inhibitor remains the cornerstone treatment in the prevention of recurrent ischaemic events following drug-eluting stents (DES) implantation. Current guidelines [1, 2] recommend a standard duration of 6 months following new-generation DES for stable coronary artery disease. A shorter duration of DAPT may be considered in patients with a high bleeding risk, and a longer duration may be considered for patients with a high ischaemic risk. New-generation DES represent devices with an improved safety profile, and are associated with lower rates of early or late stent thrombosis [3]. This raises the question what the optimal period of DAPT would be following implantation of new-generation DES.

Importantly, several randomised clinical trials have investigated optimal duration of DAPT following DES implantation. However, none of these studies were adequately powered, and some recent trials suffer from event rates that are lower than expected. Hence, we aimed to systematically review current evidence by a meta-analysis of the available randomised controlled trials (RCTs) that compared clinical outcome of short DAPT (≤6 months) with long (≥12 months) DAPT following DES implantation.

## Methods

### Search strategy and trial selection

This study was conducted according to the Preferred Reporting Items for Systematic Reviews and Meta-Analyses (PRISMA) statement [4]. We searched PubMed, EMBASE, Cochrane, international websites and meetings for RCTs that compared short (up to 6 months) with long (12 months) DAPT after DES implantation. Our systematic search on the topic of DAPT following PCI which was not limited by language, date or publication status restrictions. Detailed information regarding the full search strategy are shown in supplementary appendix 1.1, together with the PRISMA flow-chart in supplementary appendix 1.2.

### Quality assessment and risk of bias

Two individual investigators (RR and MV) independently carried out the systematic review of evidence and identified studies to be included for analysis. Three authors (RR, MV, and PS) reassessed eligibility of trials and evaluated the trials’ quality and risk of bias using the Cochrane collaboration’s tool for assessing risk of bias [5], as shown in supplementary appendix 1.3.

### Clinical outcomes

Main outcomes were all-cause death, myocardial infarction, stroke, and definite or probable stent thrombosis defined by the Academic Research Consortium (ARC) [6]. Major bleeding events were defined by trial definitions. We defined endpoints according to the ARC and adjudicated by an independent clinical event committee for each of the individual studies. Primary and secondary endpoints together with definitions of composite endpoints for each trial are shown in the supplementary appendix 1.4. Four of the included trials (EXCELLENT, RESET, ITALIC, IVUS-XPL) reported bleeding events using the TIMI criteria [7], two (SECURITY, I‑LOVE-IT 2) reported bleeding events using the Bleeding ARC (BARC) criteria [8], two reported both TIMI and BARC criteria (PRODIGY, ISAR-SAFE), and one trial (OPTIMIZE) was based on the GUSTO/REPLACE-2 criteria.

### Statistical analysis

All analyses are reported by intention to treat and based on random treatment allocation. For each outcome we calculated risk ratios (RR) and pooled estimates by the Mantel-Haenszel method [9]. Heterogeneity was estimated using the I^2^ statistic [10], with value <25% being low, a value of 25 to 50% being moderate, and a value of >75% being high. Taking into consideration the substantial differences between trials, we used random-effect models in our analysis.

Additional analyses were carried out to assess a possible impact of ST-elevation myocardial infarction (STEMI) (≥10%), acute coronary syndrome (ACS) (≥50%), first-generation DES implants (≥25%) or B2/C lesion complexity (≥60%) on clinical outcomes in short and long DAPT. To predict the true value of the RR given the fact that an additional study is published comparing short with long DAPT, we calculated the prediction interval [11]. All analyses were performed using Review Manager (RevMan) version 5.3, Copenhagen, The Nordic Cochrane Centre, The Cochrane Collaboration, 2014.

## Results

Nine RCTs (*n* = 19,099) are included in the present meta-analysis: EXCELLENT [12], RESET [13], PRODIGY [14], OPTIMIZE [15], SECURITY [16], ISAR-SAFE [17], ITALIC [18], I‑LOVE-IT 2 [19], and IVUS-XPL [20] with main characteristics shown in Tab. 1. Individual patient characteristics of trials were evenly distributed with a mean age of 64 years, 30% of patients had diabetes mellitus, and roughly 43% of patients presented with stable coronary artery disease, whereas 30% presented as low-risk ACS, as shown in supplementary appendix 1.5. Detailed procedural characteristics were also comparable with >90% of the device implants being new-generation as shown in supplementary appendix 1.6. In our analysis short DAPT ranged from 3 to 6 months with a mean duration of 5.1 months, whereas long DAPT ranged from 12 to 24 months with a mean duration of 14.6 months.

**Table 1:** Main characteristics of clinical trials evaluating short versus long duration of dual antiplatelet therapy following DES implantation

Trial	DAPT	No	Design	Inclusion criteria	Exclusion criteria	FU	MACE endpoint
EXCELLENT 2012	6 vs. 12	1443	N-I	– Stable CAD – Unstable CAD – Recent MI – Silent ischaemia – Positive functional testing	– Major bleeding event <3 months – Major surgery <2 months – MI <72 h – LVEF <25% – CTO, LM, or true bifurcation lesions – Cardiogenic shock – Dialysis	12	All-cause death, MI, stroke, ST (definite or probable), or TIMI major bleeding event
PRODIGY 2012	6 vs. 24	1970	S	– Stable CAD – ACS	– Active bleeding, history of bleeding diathesis, or prior stroke <6 months – Concomitant or foreseeable need for OAC – Planned surgery <24 months	24	All-cause death, MI, or stroke
RESET 2012	3 vs. 12	2117	N-I	– Stable CAD – ACS	– Bleeding diathesis or bleeding <3 months – Prior cerebral/peripheral arterial disease – Prior thromboembolic disease, or ST – STEMI <48 hrs – LVEF <40% – LM, CTO, or in-stent restenosis, bifurcation requiring two-stent approach – Cardiogenic shock – Severe hepatic of renal dysfunction	12	Cardiovascular death, MI, ST (definite or probable), ischaemia-driven TVR, or TIMI-major bleeding event
OPTIMIZE 2013	3 vs. 12	3119	N-I	– Stable CAD – Silent ischaemia – Low-risk ACS (unstable CAD or non-acute MI <30 days)	– STEMI – BMS non-target vessel <6 months – Prior treatment with any DES – Venous graft lesions, or in-stent restenosis	12	All-cause death, MI, stroke, major bleeding based on GUSTO/REPLACE-2
SECURITY 2014	6 vs. 12	1399	N-I	– Stable CAD – Unstable CAD – Silent ischaemia – no other DES <24 hrs – no other BMS <3 months	– Active, or significant risk of bleeding – STEMI <48 hrs or NSTEMI <6 months – LVEF <30% – Unprotected LM – Chronic kidney disease – Uncontrolled hypertension – Venous graft, or restenotic lesions	24	Cardiac death, MI, stroke, ST(definite or probable), or BARC bleeding event 2/3/5
ISAR-SAFE 2015	6 vs. 12	4005	N-I	– Patients on clopidogrel at 6 months (‑1, +2 months) after DES	– Active bleeding, bleeding diathesis, OAC or history of intracranial bleeding – MI 6 months after DES or prior ST – Major surgery <6 months – DES in the LM	15	All-cause death, MI, ST(definite or probable), stroke, TIMI major bleeding
ITALIC 2015	6 vs. 24	2031	N-I	– All clinical situations except primary PCI for acute MI or left main lesions	– Gastrointestinal or urogenital bleeding, haemorrhagic diathesis, OAC or abciximab – Non-responders to aspirin – Major (<6 weeks)/planned (<1 year) surgery – Severe liver failure	36	All-cause death, MI, stroke, TVR, TIMI major bleeding event
I-LOVE-IT 2 2016	6 vs. 12	1829	N-I	– Stable CAD – ACS	– DES implantation <1 year – LVEF <40% – Haemodynamic instability – Planned surgery <6 months – Restenotic lesions – Severe hepatic of renal dysfunction	18	All-cause death, MI, stroke, major BARC bleeding event ≥3
IVUS-XPL 2016	6 vs. 12	1400	S	– Non-emergent conditions – Stent length ≥28 mm	– Bleeding history <3 months or history of stroke, ST, PAD – STEMI within 48 hrs – Age >80 – Severe hepatic or renal dysfunction – LVEF <40%, or cardiogenic shock – LM, CTO, or in-stent restenosis, bifurcation requiring two-stent – Prior DES within 6 months – Life expectancy <1 year	12	Cardiac death, MI, stroke, or TIMI major bleeding event

*ACS* acute coronary syndrome, *BARC* bleeding academic research consortium, *BMS* bare-metal stent, *CAD* coronary artery disease, *CTO* chronic total occlusion, *DAPT* dual antiplatelet therapy, *DES* drug-eluting stent, *FU* follow-up, *LVEF* left ventricle ejection fraction, *LM* left main (coronary artery), *MACE* major adverse cardiac events, *MI* myocardial infarction, *N-I* non-inferiority, *NSTEMI* non-ST-elevation myocardial infarction, *OAC* oral anticoagulation therapy, *PCI* percutaneous coronary intervention, *PAD* peripheral artery disease, *S* Superiority, *ST* stent thrombosis, *TIMI* thrombolysis in myocardial infarction, *TVR* target vessel revascularisation

Main outcomes of the individual trials are shown in Tab. 2. Two studies (RESET, OPTIMIZE) compared 3 with 12 months of DAPT, five studies (EXCELLENT, SECURITY, ISAR-SAFE, I‑LOVE-IT 2, and IVUS-XPL) compared 6 with 12 months of DAPT, and two studies (PRODIGY, ITALIC) compared 6 with 24 months of DAPT. Potential risk of bias was considered to be generally low, albeit most of the trials were open-label (except for ISAR-SAFE), and three trials (SECURITY, ISAR-SAFE, and ITALIC) were prematurely terminated due to recruitment problems. A total of 19,099 patients were randomised and 9526 were assigned to a short regimen of DAPT (up to 6 months), and 9573 patients to long DAPT (at least 12 months).

**Table 2:** Clinical outcomes of clinical trials evaluating short versus long duration of dual antiplatelet therapy following DES implantation

Trial	Short vs. long DAPT	MACE/MACCE endpoint short vs. long	*p*-value	Major bleeding event short vs. long	*p*-value	Definite or probable ST short vs. long	*p*-value
EXCELLENT	6 vs. 12	8.0% vs. 8.5%^1^ HR 0.94 (0.65 to 1.35)	0.72	0.3% vs. 0.6%^2^ HR 0.50 (0.09 to 2.73)	0.42	0.9% vs. 0.1% HR 6.02 (0.72 to 50)	0.10
PRODIGY	6 vs. 24	10.0% vs 10.1%^3^ HR 0.98 (0.74 to 1.29)	0.91	1.9% vs. 3.4%^4^ HR 0.56 (0.32 to 0.98)	0.037	1.5% vs. 1.3% HR 1.15 (0.55 to 2.44)	0.70
RESET	3 vs. 12	4.7% vs. 4.7%^5^ RD 0.0% [−2.5 to 2.5]	0.84	0.2% vs. 0.6%^6^ RD −0.4% [−0.9 to 0.1]	0.16	0.2% vs. 0.3% RD −0.1% [−0.5 to 0.3]	0.65
OPTIMIZE	3 vs. 12	6.0% vs. 5.8%^7^ HR 1.03 (0.77 to 1.38)	0.84	0.6% vs. 0.9%^8^ HR 0.71 (0.32 to 1.60)	0.41	0.8% vs. 0.8% HR 1.08 (0.49 to 2.36)	0.86
SECURITY	6 vs. 12	4.5% vs. 3.7%^9^ RD 0.8% [−2.4 to 1.7]	0.47	0.6% vs. 1.1%^10^ RD −0.5% [−1.4 to 0.4]	0.28	0.3% vs. 0.4% RD −0.1% [−0.7 to 0.4]	0.69
ISAR-SAFE	6 vs. 12	1.5 vs. 1.6^11^ HR 0.91 (0.55 to 1.50)	0.70	1.0% vs. 2.0%^12^ HR 0.50 (0.29 to 0.85)	0.01	0.3% vs. 0.2% HR 1.25 (0.33 to 4.65)	0.74
ITALIC	6 vs. 24	1.6% vs. 1.5%^13^ HR 1.07 (0.51 to 2.22)	0.85	0.0% vs. 3.0%^14^ NA	–	3.0% vs. 0.0% N/A	–
I-LOVE-IT 2	6 vs. 12	7.2% vs. 6.4%^15^ NA	0.53	0.7% vs 1.2%^16^ NA	0.21	1.1% vs. 0.8% N/A	0.33
IVUS-XPL	6 vs. 12	2.2% vs. 2.1%^17^ HR 1.07 (0.52 to 2.22)	0.85	0.7% vs. 1.0%^18^ HR 0.71 (0.23 to 2.25)	0.56	0.3% vs. 0.3% HR 1.00 (0.14 to 7.11)	0.99

^1^MACCE (1-year): any death, MI, stroke, or any revascularisation; ^2^TIMI major bleeding event; ^3^MACCE (2-year): any death, MI, stroke; ^4^BARC 3/5 major bleeding event; ^5^MACE (1-year): cardiovascular death, MI, ST (def. or prob.), ischaemia-driven TVR, or TIMI bleeding; ^6^TIMI major bleeding event; ^7^MACCE (1-year): any death, MI, stroke, or major bleeding; ^8^REPLACE-GUSTO; ^9^MACCE (1-year): cardiac death, MI, stroke, ST (def. or prob.), or BARC 3/5; ^10^BARC 3/5 major bleeding event; ^11^MACCE (15-months): any death, MI, ST (def. or prob.), stroke, or TIMI major bleeding; ^12^BARC 2/3/5 bleeding event; ^13^MACCE (1-year): any death, MI, stroke, TVR, or TIMI major bleeding; ^14^TIMI major bleeding event; ^15^MACCE (1-year): any death, MI, stroke, major bleeding BARC ≥3; ^16^BARC 3/4/5 major bleeding event; ^17^MACCE (1-year): cardiac death, MI, stroke, TIMI major bleeding; ^18^TIMI major bleeding event

*BARC* bleeding academic research consortium, *DAPT* dual antiplatelet therapy, *HR* hazard ratio, *MACE* major adverse cardiac events, *MACCE* major adverse cardiac and cerebrovascular events, *MI* myocardial infarction, *N/A* not available, *RD* risk difference, *ST* stent thrombosis, *TIMI* thrombolysis in myocardial infarction, *TVR* target vessel revascularisation

## Primary analysis of short versus long DAPT after DES implantation

The primary outcome for each individual major clinical trial comparing short versus long DAPT are summarised in Fig. 1. This figure shows that short DAPT in patients with low ischaemic risk leads to a significant reduction in major bleeding events, whereas all-cause mortality, myocardial infarction, stent thrombosis, and stroke were similar.

**Fig. 1:**
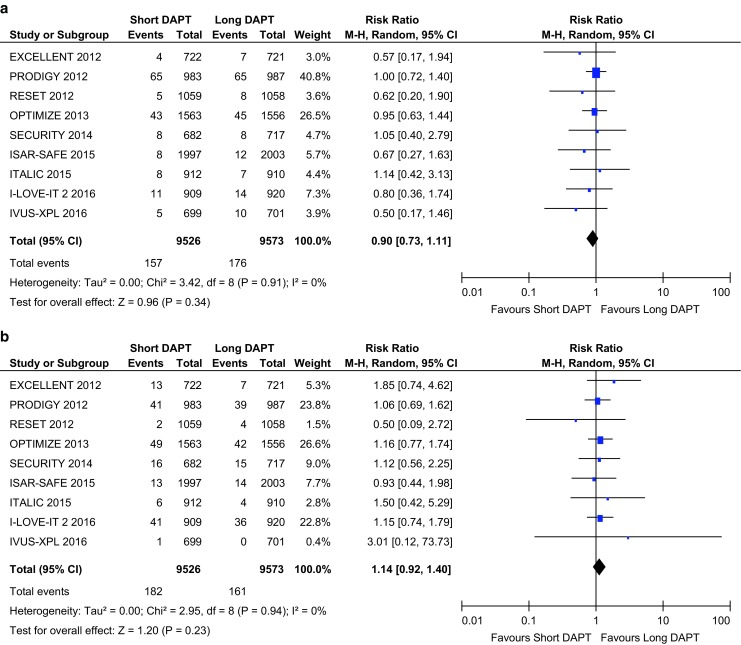
Forest plots reporting of pooled risk ratios with 95% confidence intervals of **a** all-cause mortality; **b** myocardial infarction; **c** probable or definite stent thrombosis; **d** stroke; **e** BARC ≥3 major bleeding event. *Horizontal lines* represent the 95% confidence interval, *the square* represents the risk ratio of each individual study, the *diamond* represents the pooled risk ratios and the overall effect. *DAPT* dual antiplatelet therapy, *CI* confidence interval, *M-H* Mantel-Haenszel

**Fig. 1:**
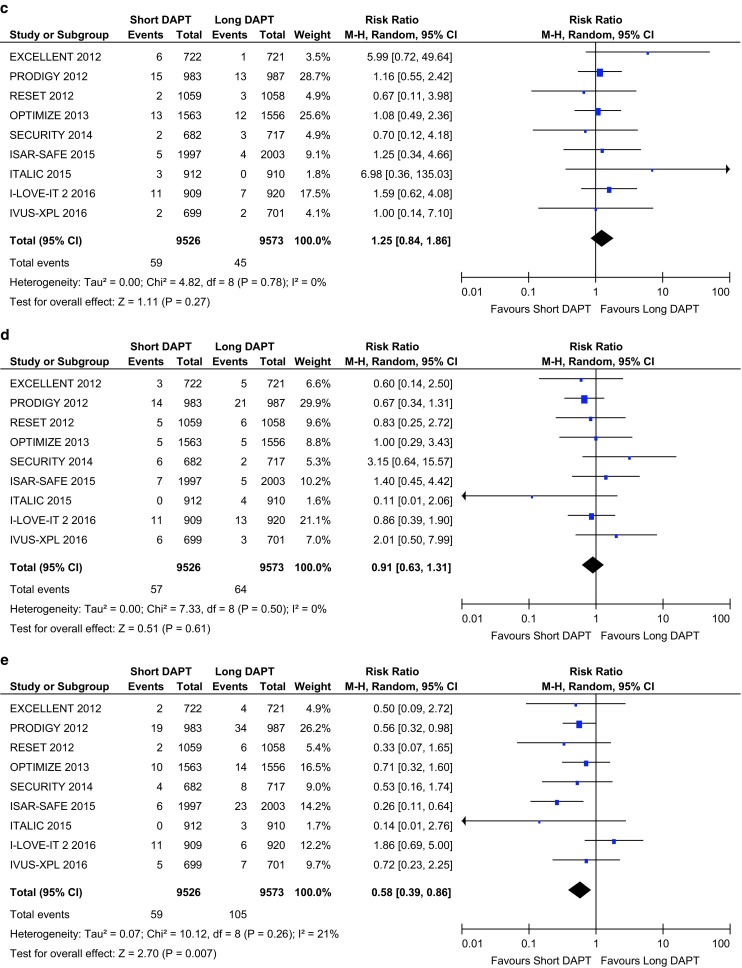
continued

### All-cause mortality

Death rate due to all-causes in patients who underwent PCI with DES implantation was not statistically different for a short duration of DAPT when compared with longer duration of DAPT (Fig. 1a).

### Myocardial infarction

Myocardial infarction was numerically higher in short DAPT when compared with long DAPT, without reaching the level of statistical significance (Fig. 1b).

### Stent thrombosis

The overall rate of definite or probable stent thrombosis was 0.56%. Differences among the rate of stent thrombosis in short versus long DAPT did not reach the level of statistical significance (Fig. 1c).

### Stroke

We did not detect any differences in cerebrovascular accidents for short DAPT when compared with long DAPT (Fig. 1d).

### Major bleeding events

The overall rate of major bleeding events was 0.86%. Major bleeding rates were available for all trials included in this meta-analysis, although the event rates reported by four trials were defined based on TIMI criteria, two were based on BARC criteria, and one was based on modified REPLACE-2/GUSTO criteria. Short duration of DAPT was associated with a significant reduction in the risk of major bleeding events (event rate 0.62% vs. 1.10%, RR 0.58, 95% CI 0.39 to 0.86, *p* < 0.007, I^2^ = 21%, see Fig. 1e).

## Sensitivity analysis

The results obtained with sensitivity analyses were highly consistent, and did not modify any of our main findings. We carried out additional analyses to investigate the impact of studies with a relatively high number of STEMI (≥10%), ACS (≥50%), first-generation DES (≥25%), and complex lesions (≥60%).

## Prediction interval

Regarding the primary analysis comparing short with long DAPT, the likely values of the true RR were calculated considering that a new study will be published. Most endpoints (all-cause mortality, myocardial infarction, stent thrombosis, and stroke) did not show considerable heterogeneity and therefore the 95% CI could be interpreted as a prediction interval. Regarding major bleeding events, however, the 95% prediction interval is considered to range from 0.31 to 1.06.

## Discussion

In this meta-analysis, which included more than nineteen thousand randomised patients scheduled for PCI with DES implantation, we evaluated clinical outcomes of short versus long DAPT. Several trials have been conducted to evaluate DAPT following stent implantation. However, the optimal duration of DAPT remains controversial, and none of these trials [12–19] were adequately powered.

Our findings demonstrate that (1) short DAPT, when compared with long DAPT, was associated with a reduction of roughly 50% in major bleeding events, and (2) short DAPT was not associated with an apparent increase of all-cause death, myocardial infarction, stent thrombosis, or stroke. Our findings support the use of short DAPT in stable patients, as we believe that prolongation of DAPT regimens will result in a detrimental increase in bleeding events. High-risk patients on the other hand, may benefit from a longer duration of DAPT and are beyond the scope of this analysis as our primary analysis is not powered for this particular subgroup.

Several meta-analyses evaluated short versus long DAPT. The findings of our meta-analysis differ from the previous meta-analyses that were carried out with fewer patients in the short DAPT regimen [21–23] or with higher heterogeneity [21, 24–26]. Consistent with our findings, previous meta-analyses have shown that short DAPT is associated with lower rates of major bleeding events when compared with long DAPT [22, 23]. We should emphasise that bleeding events due to DAPT are time-independent, meaning that the overall risk of bleeding will continue to rise with longer durations of DAPT. In contrast to previous meta-analyses [22, 27], short DAPT did not lead to an increased rate of ischaemic events in our analysis, which may be due to the fact that our analysis contains a considerable number of stable patients and may not be valid for high-risk patients. For instance, a subgroup that may particularly benefit from longer durations of DAPT are stable patients with a history of myocardial infarction, as demonstrated by a recent meta-analysis [28].

Nearly two decades ago, DAPT was shown superior in ACS in terms of efficacy when compared with aspirin alone [29]. A landmark analysis of CURE revealed that the majority of ischaemic events are prevented within the first 3 months (20/1,000 patients treated). After this period, this protective effect is substantially attenuated (2/1,000 patients treated) and the rate of significant bleeding events may become more important than the number of prevented ischaemic events. What is complicating our interpretation, however, is that this pivotal study was conducted in the BMS era and a considerable number of patients with ACS were being treated conservatively. A recent meta-analysis [30] demonstrated that a short duration of DAPT (3 months) in ACS was associated with higher rates of myocardial infarction and stent thrombosis. Some clinicians consider major bleeding events less deleterious when compared with acute ischaemic events. Noteworthy, post-discharge bleeding events are still common and directly associated with increased mortality. In fact, post-discharge major bleeding events should not be underestimated as the effect size is sometimes even greater than that of post-discharge myocardial infarction [31]. Opposing this statement is a substudy of ADAPT-DES [32] that evaluated the impact of stent thrombosis, myocardial infarction that is not stent-related, and clinically relevant bleeding events on mortality, and found that the risk of mortality was increased in both post-PCI ischaemic and bleeding events. Remarkably, early stent thrombosis and very late spontaneous myocardial infarction were associated with the highest risk of mortality, whereas clinically significant bleeding events and myocardial infarction that is not stent-related were associated with a similar but lower risk of mortality.

## Limitations

Some limitations in the present meta-analysis should be acknowledged. First, a meta-analysis of individual patient data would allow us to directly analyse parameters influencing outcomes following short or long DAPT. We believe, however, that we can reliably evaluate the topic of interest with our approach. Second, the reported event rate (death, myocardial infarction and stroke) in some trials was >10% (PRODIGY), whereas the event rate (death, myocardial infarction, target vessel revascularisation and bleeding) of others was as low as 1.5% (ITALIC), and may therefore be indicative for underreporting in some of the studies in this meta-analysis. The accuracy of our results reflects the quality of the included studies. Some variance was introduced by clinical endpoint definitions, especially in bleeding event endpoints. The conservative definitions for major bleeding events that some trials used may have underestimated the rate of major bleeding events as we believe should be evaluated by the BARC criteria. Even though endpoint definitions were not uniform, we suppose this did not modify our overall conclusion. Finally, in most trials clopidogrel was used as a P2Y12 inhibitor. Considering the frequent use of more potent P2Y12 inhibitors in routine clinical practice, the incidence of major bleeding events in our analysis may be underestimated.

## Conclusions

A short regimen of DAPT after PCI using a DES implantation seems to provide a significant reduction in major bleeding events without compromising ischaemic events in patients with stable coronary artery disease. To drive patient-tailored antiplatelet therapy, the delicate balance of bleeding complications as opposed to the risk of ischaemic events should be investigated in patient-specific subgroups.

## Caption Electronic Supplementary Material

Search strategy, PRISMA-flow chart, risk of bias evaluation, and additional trial characterics. Supplementary results include additional analysis to investigate the impact of studies with STEMI (≥10%), ACS (≥50%), first-generation DES (≥25%), and complex lesions (≥60%)
