# Supplementary material for: Short versus long duration of dual antiplatelet therapy following drug-eluting stents: a meta-analysis of randomised trials
Source: Neth Heart J. 2018 Mar 14;26(5):242–51. doi: 10.1007/s12471-018-1104-6 (PMC5910311; doi:10.1007/s12471-018-1104-6)

**Supplementary Appendix**

**1 Supplementary Methods**

**1.1 Search Strategy**

**1.2 PRISMA-Flow Diagram**

**1.3 Risk of Bias of Major Trials.**

**1.4 Supplementary table 1. Clinical Endpoints in Major Trials evaluating DAPT.**

**1.5 Supplementary table 2. Individual Patient Characteristics of Major Trials evaluating DAPT**

**1.6 Supplementary table 3. Procedure Characteristics of Major Trials evaluating DAPT**

**2 Supplementary Analysis**

**2.1 ≥10% STEMI patients**

**2.2 high numbers of patients with ACS (≥50%)**

**2.3 first-generation DES implants (≥25%)**

**2.4 high number of complex lesions (≥60%)**

**1 Supplementary Methods**

**1.1 Search strategy**

A systematic review of evidence was performed using the keywords “dual antiplatelet therapy”, “aspirin”, “acetylsalicylic acid”, “clopidogrel”, “thienopyridine”, “P2Y12-inhibitor”, “ADP-inhibitor”, “drug-eluting stent”, and “trial”. Regarding this meta-analysis, we used the following exclusion criteria: clinical outcomes based on observational data, patients without documented coronary artery disease, percutaneous coronary intervention without DES implantation, or studies using DAPT-regimens inappropriate for the present comparison of short versus long DAPT. Our search was not limited by any language-, date-, or publication status restrictions.

On April 9^th^, 2017, we performed a search not limited by language or date, using the primary algorithm: ((((dual antiplatelet therapy OR aspirin OR acetylsalicylic acid OR clopidogrel OR thienopyridine OR P2Y12-inhibitor OR ADP-inhibitor)) AND drug-eluting stent) AND trial)”. We found the 1873 results using PubMed=535, Cochrane=572, and EMBASE=766.

Furthermore, we searched for presentations on major cardiovascular meetings and searched for additional studies at important websites:

<https://clinicaltrials.gov/>

<http://clinicaltrialresults.org/>

<https://www.acc.org>

<https://www.tctmd.com/>

<https://www.pcronline.com/>

After removing duplicates we identified 1362 articles that were considered potentially relevant.

**1.2 PRISMA-flow diagram**

Articles excluded (n = 1037 )

Non-trial studies (n = 288 )

Non-relevant (n = 1069 )

Articles screened for eligibility (n = 1362 )

Articles after duplicates removed
(n = 1362 )

No additional studies were identified through other sources
(n = 0 )

Potential articles identified through database searching
(n = 1873 )

## Identification

## Screening

Full-text articles excluded:

Non-randomized (n = 14 )

Wrong intervention (n = 8 )

No DES/PCI (n = 6 )

Full-text articles assessed for eligibility
(n = 37 )

## Eligibility

Trials included in quantitative synthesis
(Randomized trials n = 9 )

(Participants n = 15870 )

## Included

*From:*Moher D, Liberati A, Tetzlaff J, Altman DG, The PRISMA Group (2009). *P*referred *R*eporting *I*tems for *S*ystematic Reviews and *M*eta-*A*nalyses: The PRISMA Statement. PLoS Med 6(7): e1000097. doi:10.1371/journal.pmed1000097

**For more information, visit** [**www.prisma-statement.org**](http://www.consort-statement.org/)**.**

**1.3 Risk of bias of included Major Trials**

| **Trial** | **Random sequence generation** (selection bias) | **Allocation concealment** (selection bias) | **Blinding of participants and healthcare providers** (performance bias) | **Blinding of outcome assessment** (detection bias) | **Intention to treat**  (attrition bias) | **Incomplete outcome data** (attraction bias) | **Selective reporting**  (reporting bias) | **Any other potential bias** |
| --- | --- | --- | --- | --- | --- | --- | --- | --- |
| RESET | 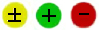 | 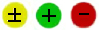 | 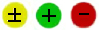 | 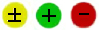 | 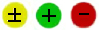 | 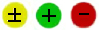 | 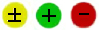 | 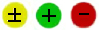 |
| PRODIGY | 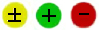 | 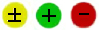 | 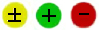 | 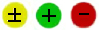 | 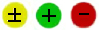 | 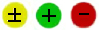 | 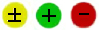 | 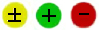 |
| EXCELLENT | 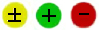 | 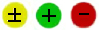 | 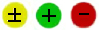 | 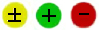 | 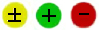 | 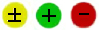 | 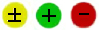 | 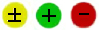 |
| OPTIMIZE | 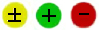 | 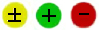 | 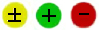 | 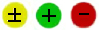 | 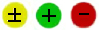 | 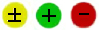 | 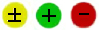 | 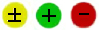 |
| SECURITY | 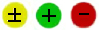 | 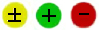 | 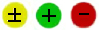 | 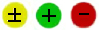 | 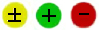 | 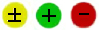 | 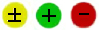 | 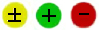 |
| ISAR-SAFE | 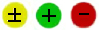 | 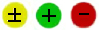 | 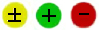 | 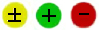 | 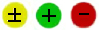 | 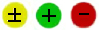 | 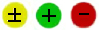 | 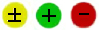 |
| ITALIC | 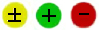 | 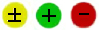 | 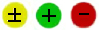 | 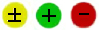 | 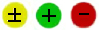 | 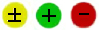 | 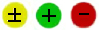 | 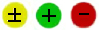 |
| I-LOVE-IT 2 | 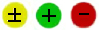 | 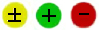 | 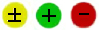 | 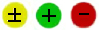 | 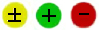 | 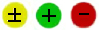 | 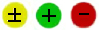 | 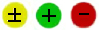 |
| IVUS-XPL | 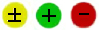 | 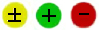 | 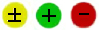 | 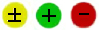 | 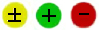 | 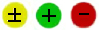 | 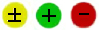 | 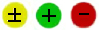 |
| 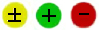; low risk of bias 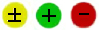 ; high risk of bias | | | | | | | | |

All trials randomly assigned patients with decent allocation concealment (low risk of selection bias), were open-label (high risk of performance bias) except for the double-blind ISAR-SAFE (low risk of performance bias), and were blinded for outcome (adjudicated clinical events by an independent event committee). All trials were analyzed by intention-to-treat, and reported events and lost-to-follow-up throughout the follow-up period. Three trials were prematurely terminated due to poor enrolment and/or lower-than-expected event rates (high risk of attraction bias, or other bias): SECURITY stopped at 1399 participants (2740 planned) , ISAR-SAFE stopped at 4005 participants, (6000 planned), and ITALIC stopped at 2031 participants (2475 planned)

**1.4 Clinical Endpoints in Major Clinical Trials evaluating DAPT after DES implantation.**

| **Trial** | **Primary Endpoint** | **Sub-analysis** | **Secondary Endpoints** | **MACE-endpoint** |
| --- | --- | --- | --- | --- |
| EXCELLENT  6 vs. 12 | - 1-year - TVF (cardiac death, MI, ischemia-driven target vessel revascularization) | - Age < 65 - ACS yes/no - DM yes/no - LVEF <50 yes/no - Bifurcation - Type of DES - Multi-stenting | - All-cause death - Cardiac death - Myocardial infarction (>3UNL) - Stroke (neurologist and imaging) - Stent thrombosis (definite, prob, pos) - TLR - Any bleeding - TIMI major bleeding | All-cause death,  MI,  stroke,  ST(definite or probable),  TIMI-major bleeding |
| PRODIGY  6 vs. 24 | - **2- year,** composite of   all cause death, MI, CVA. | - Age < 65 - Gender - DM - Type of DES (BMS, DES) - SCAD, Unstable AP - Single / multiple lesion(s) treated - Simple / complex lesions - Creat <60 | - All-cause death - Cardiovascular death - Myocardial infarction - Cerebrovascular accident - Stent thrombosis (definite, prob, pos) - Any bleeding (TIMI/BARC) - Major bleeding (TIMI/BARC) - Major bleeding BARC 2/3/5 | All-cause death,  MI,  cerebrovascular accident |
| RESET  3 vs. 12 | - 1-year, composite of - **cardiovascular death**, MI, ST, ischemia-driven target-vessel revascularization, **bleeding** | - DM - ACS - Short DES - Long DES | - All-cause death - Cardiovascular death - Myocardial infarction - Cerebrovascular accidents - Stent thrombosis (definite, prob, pos) - Target vessel revascularization - Non-target vessel revascularization - Bleeding: major or minor | Cardiovascular death,  MI,  ST(definite or probable),  ischemia-driven TVR  TIMI-major bleeding |
| OPTIMIZE  3 vs. 12 | - 1-year, composite of - All-cause death, MI, stroke, **major bleeding (GUSTO/REPLACE-2)** | - DM - ACS - Bifurcation - Lesion complexity - Single / multiple lesion(s) treated - Simple / complex lesions - Size large/small - In-stent restenosis | - All-cause death - Cardiac death - Myocardial infarction - Target lesion revascularization - Target vessel revascularization - Stent thrombosis (definite, prob, pos) - Any bleeding - Major bleeding | All-cause death,  MI,  stroke,  major bleeding (GUSTO/REPLACE-2 criteria) |
| SECURITY  6 vs. 12 | - 1-year, composite of - **cardiac** death, MI, ST, BARC3,5 major bleeding | predictors of the primary endpoint†   - Age > 75 y - DM - Stent type - Mean DES length - Mean stent size | - Cardiovascular death - Myocardial infarction - Cerebrovascular accidents - Stent thrombosis (definite, prob, pos) - Any bleeding BARC 2/3/5 - Major bleeding BARC | Cardiac death,  MI,  stroke,  ST(definite or probable)  bleeding BARC2/3/5 |
| ISAR-SAFE  6 vs. 12 | - **15-months**, composite of - All-cause death, myocardial infarction, stent thrombosis, stroke, and TIMI-major bleeding | - Age < 67 - Gender male/female - DM yes/no - ACS yes/no - Complex lesion yes/no - LVEF < 55% - Premature DAPT cessation - Current smoking | - All-cause death - Myocardial infarction - Cerebrovascular accidents - Stent thrombosis (definite, prob) - Any bleeding BARC 2/3/5 - TIMI minor/ major | All-cause death,  MI,  ST(definite or probable),  stroke,  TIMI-major bleeding |
| ITALIC  6 vs. 24 | - 1 year, composite of - All-cause death, MI, **urgent TVR**, stroke, TIMI-major bleeding. | - ACS vs non-ACS   - Primary endpoint   - All-cause, or cardiac death   - MI,   - TVR,   - Minor or major bleeding | - All-cause death - Cardiac death - Myocardial infarction - Cerebrovascular accidents - Stent thrombosis (definite, prob) - TIMI minor/ major | All-cause death,  MI,  stroke,  TVR,  TIMI-major bleeding |
| I-LOVE-IT 2  6 vs. 12 | - 1-year - TLF (cardiac death, MI, clinically indicated target vessel revascularization) | - Age < 65 - Gender male/female - BMI <30 - Current smoker - Unstable angina - Multivessel, no of DES, CTO, bifurcation, small / long | - All-cause death - Cardiac death - Myocardial infarction - Cerebrovascular accidents - Stent thrombosis (definite, prob) - TLR/ TVR - BARC>3 Major bleeding | All-cause death,  MI,  Stroke,  Major bleeding BARC≥3 |
| IVUS-XPL  6 vs. 12 | - 1-year - **cardiovascular death**, MI, stroke, TIMI-major bleeding. | - Age < 65 - Gender - Hypertension - DM - Dyslipidemia - Current smoker - ACS yes/no - Multivessel - Target vessel - Reference vessel diameter - Use of IVUS | - All-cause death - Cardiac death - Myocardial infarction - Repeat revascularization - Stent thrombosis (definite, prob) - TIMI-major bleeding | Cardiac death,  MI,  stroke,  TIMI-major bleeding |

† analyzed as cox regression multivariate analysis

ACS = Acute Coronary Syndrome, AP = Angina Pectoris, BARC = Bleeding Academic Research Consortium, BMI = Body-Mass Index, CTO = Chronic Total Occlusion, DES = Drug-eluting Stent, DM = Diabetes mellitus, IVUS = Intravascular ultrasound, MI = Myocardial Infarction, SCAD = Stable Coronary Artery Disease, ST = Stent Thrombosis, TIMI = Thrombolysis in Myocardial Infarction, TLR = Target-Lesion Failure, TVF = Target-Vessel Failure.

1.5. Individual Patient Characteristics of trials evaluating short- versus long duration of dual antiplatelet therapy following DES-implantation.

| **Trial** | **DAPT** (months) | **No.** (n) | **Male**  (n%) | **Age**  (mean±Sd) | **HT**  (n%) | **DM**  (n%) | **Dyslipidemia**  (n%) | **Current Smoker** (n%) | **BMI**  (mean±Sd) | **LVEF**  (mean±Sd) | **RI**  (n%) | **Prior MI** (n%) | **SCAD** (n%) | **Low-risk ACS** (n%) | **ACS**  (n%) | **STEMI**  (n%) | **MVD** (n%) |
| --- | --- | --- | --- | --- | --- | --- | --- | --- | --- | --- | --- | --- | --- | --- | --- | --- | --- |
| EXCELLENT | 6 | 722 | 65.1 | 63.0±9.6 | 72.7 | 37.7 | 75.2 | 27.4 | 24.9±3.1 | 61.0±9.6 | NA | 6.5 | 48.9 | 48.5‡ | 51.1 | 2.6 | 51.9 |
|  | 12 | 721 | 63.9 | 62.4±10.4 | 73.8 | 38.6 | 76.3 | 25.8 | 25.1±3.0 | 61.6±9.4 | NA | 3.7 | 48.0 | 48.4‡ | 52.0 | 3.6 | 52.0 |
| PRODIGY | 6 | 983 | 76.0 | 67.9±11 | 70.4 | 23.7 | 53.4 | 25.1 | 26.6 (24.6-29.4)§ | 50.0 (43.3-60.0) § | NA | 26.2 | 25.4 | 18.5 | 55.4 | 33.3 | 66.0 |
|  | 24 | 987 | 77.4 | 67.8±11 | 73.0 | 24.7 | 56.0 | 22.5 | 26.7 (24.8-29.3) § | 55.0 (45.3-60.0) § | NA | 27.3 | 26.0 | 18.5 | 56.1 | 32.5 | 65.2 |
| RESET | 3 | 1059 | 64.4 | 62.4±9.4 | 62.3 | 29.8 | 57.7 | 25.2 | 25.0±3.2 | 64.2±9.4 | NA | 1.8 | 44.5 | 40.8 | 55.5 | 14.7 | 43.1 |
|  | 12 | 1058 | 62.9 | 62.4±9.8 | 61.4 | 28.8 | 59.9 | 22.8 | 24.9±3.1 | 63.9±9.4 | NA | 1.6 | 46.3 | 39.9 | 53.7 | 13.8 | 42.9 |
| OPTIMIZE | 3 | 1563 | 63.5 | 61.3±10.4 | 86.4 | 35.4 | 63.2 | 18.6 | NA | NA | 7.4 | 34.6 | 59.8 | 26.2 | 31.6 | 0 | NA |
|  | 12 | 1556 | 63.1 | 61.9±10.6 | 88.2 | 35.3 | 63.7 | 17.3 | NA | NA | 5.8 | 34.8 | 58.6 | 26.8 | 32.3 | 0 | NA |
| SECURITY | 6 | 682 | 77.6 | 64.9±10.2 | 74.5 | 30.4 | 65.4 | 20.5 | NA | 56.3±8.7 | NA | 21.2 | 61.6 | 38.4 | 38.4 | 0 | 43.8 |
|  | 12 | 717 | 76.8 | 65.5±10.1 | 71.1 | 31.4 | 60.8 | 24.4 | NA | 56.6±8.2 | NA | 20.1 | 61.6 | 38.4 | 38.4 | 0 | 40.8 |
| ISAR-SAFE | 6 | 1998 | 80.7 | 67.2 (59-73) § | 90.1 | 24.8 | 87.5 | 14.6 | 27.2 (24.9-30.1) § | NA | NA | 25.9 | 48.6 | 21.5 | 18.3 | 7.9 | 61.3 |
|  | 12 | 2007 | 80.5 | 67.2 (59-73) § | 91.5 | 24.2 | 87.4 | 15.3 | 27.5 (24.9-30.4) § | NA | NA | 24.5 | 47.8 | 21.9 | 18.4 | 8.3 | 61.8 |
| ITALIC | 6 | 926 | 79.2 | 61.7±10.9 | 65.2 | 36.3 | 67.1 | NA | 27.0±4.6 | NA | 3.1 | 14.7 | 41.1 | 15.7 | 7.4 | 0.1 | NA |
|  | 24 | 924 | 80.8 | 61.5±11.1 | 64.7 | 37.8 | 67.1 | NA | 27.1±4.7 | NA | 2.7 | 15.6 | 41.5 | 16.4 | 7.4 | 0.3 | NA |
| I-LOVE-IT-2 | 6 | 909 | 67.2 | 60.4±10.2 | 61.0 | 23.2 | 25.3 | 36.6 | 25.1±3.1 | 60.8±8.4 | NA | 17.2 | 14.3 | 58.0 | 24.7 | 11.3 | 24.2 |
|  | 12 | 920 | 68.2 | 60.0±10.0 | 64.8 | 22.1 | 23.4 | 38.3 | 25.3±3.0 | 60.3±8.2 | NA | 15.8 | 15.1 | 56.5 | 24.4 | 10.7 | 22.7 |
| IVUS-XPL | 6 | 699 | 67.2 | 63±9 | 63 | 35.6 | 67.6 | 24.5 | 24.8±3.1 | 62.3±10.2 | NA | 4.9 | 50.9 | 33.9 | 49.1 | NA | 67.2 |
|  | 12 | 701 | 70.5 | 64±9 | 65 | 36.6 | 65.0 | 23.5 | 24.6±3.0 | 63.1±9.7 | NA | 4.0 | 51.1 | 32.9 | 48.9 | NA | 69.9 |

† Unstable Angina (troponin-negative ACS), ‡ Unstable Angina and NSTEMI. § reported as mean – range.

ACS = Acute Coronary Syndrome, BMI = Body Mass Index, DAPT = Dual Antiplatelet Therapy, DM = Diabetes Mellitus, HT = Hypertension, LVEF = Left Ventricle Ejection Fraction, MI = Myocardial Infarction, MVD = Multivessel Disease, NA = Not Available, SCAD = Stable Coronary Artery Disease, STEMI = ST segment elevation myocardial infarction, RI = Renal Insufficiency.

1.6. Procedure Characteristics of Trials evaluating short- versus long duration of dual antiplatelet therapy following DES-implantation.

| **Trial** | **DAPT** (months) | **DAPT Adherence**  (%) | **Lesion B2/C**  (n%) | **LM**  (n%) | **LAD**  (n%) | **Graft**  (n%) | **DES-types**  (generation, n%) | **No. of DES per patient** (mean±sd) | **DES length per patient**  (mean±sd) |
| --- | --- | --- | --- | --- | --- | --- | --- | --- | --- |
| EXCELLENT | 6 | Aspirin 99.4  Clopidogrel 98.7 | 52.8 | 0 | 50.6 | 0 | 2^nd^-EES, 75  1^st^-SES, 25 | 1.6±1.0 | 27.8±13.0† |
|  | 12 | Aspirin 98.7  Clopidogrel 99.6 | 53.8 | 0 | 49.0 | 0 | 2^nd^-EES, 75  1^st^-SES, 25 | 1.6±0.9 | 28.3±13.7†‡ |
| PRODIGY | 6 | Aspirin 99.1  Clopidogrel 83.6 | 67.7 | 5.7 | 52.7 | 1.7 | 2nd-EES, 25  2nd-ZES, 25  1st-PES, 25  3rd-BMS, 25 | 1.8±1.2 | 30 (20-48) |
|  | 24 | Aspirin 99.0  Clopidogrel 98.3 | 65.1 | 5.6 | 52.5 | 2.3 | 2nd-EES, 25  2nd-ZES, 25  1st-PES, 25  3rd-BMS, 25 | 1.9±1.3 | 30 (20-48) |
| RESET | 3 | NA | 67.9 | 0 | 52.7 | NA | 2^nd^-ZES 100 | NA | 22.7±10.1 |
|  | 12 | NA | 69.2 | 0 | 53.8 | NA | 2^nd^-ZES, 41.5  2^nd^-EES, 30.0  1^st^-SES, 28.5 | NA | 22.7±10.7 |
| OPTIMIZE | 3 | Aspirin 99.5  Clopidogrel 99.8 | 37.0‡ | 1.2 | 47.9 | 0 | 2^nd^-ZES, 100 | 1.6±0.8 | 32.8±19.8 |
|  | 12 | Aspirin 99.3  Clopidogrel 99.7 | 37.4‡ | 1.5 | 46.6 | 0 | 2^nd^-ZES, 100 | 1.6±0.8 | 32.7±20.0 |
| SECURITY | 6 | Aspirin 99.0  Clopidogrel 98.1 Prasugrel 0.3  Ticagrelor 0.6 | 21.1 | 0 | 43.0 | 0 | 2^nd^-ZES, 42  2^nd^-BES, 33  2^nd^-EES, 21 | 1.6±0.9 | 19.1±7.2 |
|  | 12 | Aspirin 99.4  Clopidogrel 99.3  Prasugrel 0.1  Ticagrelor 0.3 | 21.0 | 0 | 44.0 | 0 | 2^nd^-ZES, 40  2^nd^-BES, 35  2^nd^-EES, 20 | 1.6±0.9 | 19.0±7.2 |
| ISAR-SAFE | 6 | NA | 42.3 | 0.5 | 39.8 | 1.5 | 2^nd^-EES, 48§  2^nd^-SES, 16  2^nd^-ZES, 15  2^nd^-BES, 9 | 1.7±1.0 | 28 (18-43) |
|  | 12 | NA | 45.5 | 0.2 | 40.6 | 1.3 | 2^nd^-EES, 48§  2^nd^-SES, 16  2^nd^-ZES, 15  2^nd^-BES, 9 | 1.7±1.0 | 28 (18-43) |
| ITALIC | 6 | Aspirin 100%  Clopidogrel 98.9% | NA | NA | 73.4 | 6.5 | 2^nd^-EES, 100 | 1.7±1.0 | 38.6±25.6 |
|  | 24 | Aspirin 100%  Clopidogrel 98.4% | NA | NA | 72.3 | 4.3 | 2^nd^-EES, 100 | 1.7±1.0 | 37.8±26.1 |
| I-LOVE-IT-2 | 6 | Aspirin 99.7%  Clopidogrel 99.7% | 83.6 | 1.9 | 45.9 | NA | 2^nd^-SES 100 | 1.7±0.9 | 41.0±25.1 |
|  | 12 | Aspirin 98.6%  Clopidogrel 98.1% | 83.4 | 1.7 | 45.3 | NA | 2^nd^-SES 100 | 1.7±0.9 | 41.2±24.6 |
| IVUS-XPL | 6 | Aspirin 98.9%  Clopidogrel 98.9% | NA | 0 | 54.8 | NA | 2^nd^-EES 100 | 1.6±0.9 | 46.5±19.7 |
|  | 12 | Aspirin 99.5%  Clopidogrel 99.5% | NA | 0 | 55.7 | NA | 2^nd^-EES 100 | 1.6±0.8 | 48.2±20.2 |

† DES-length per lesion, ‡ lesion Type C, §other 1st-generation DES: SES 8%, 1st-PES 3%.

BMS = Bare-metal Stent, DAPT = Dual Antiplatelet Therapy, DES = Drug-Eluting Stent, EES = Everolimus-eluting Stent, LAD = Left Anterior Descending artery, LM = Left Main artery, NA = Not Available, PES Paclitaxel-eluting Stent, SES = Sirolimus-eluting Stent, ZES = Zotarolimus-eluting Stent.

**2 Supplementary Analysis**

**2.1 Additional Analysis, ≥10% STEMI**

All-cause mortality


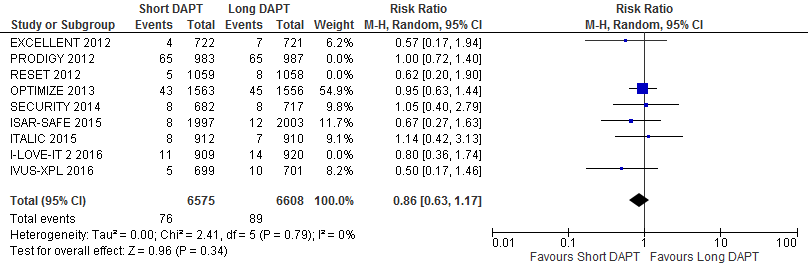


Myocardial infarction


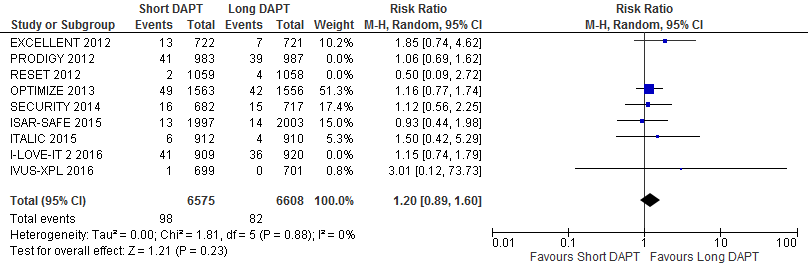


Stent Thrombosis


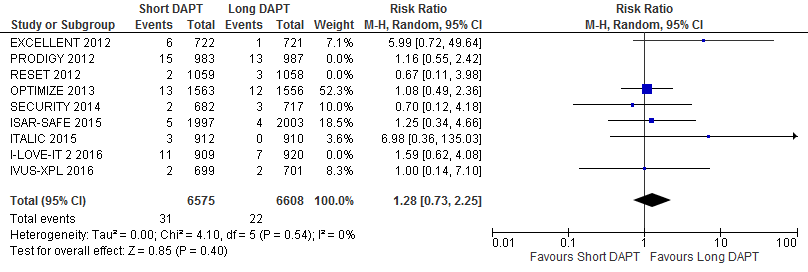


Stroke


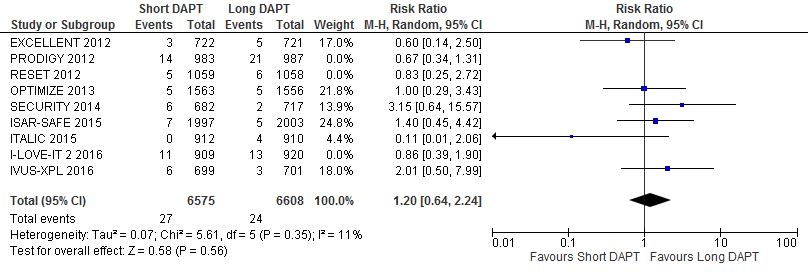


Major bleeding


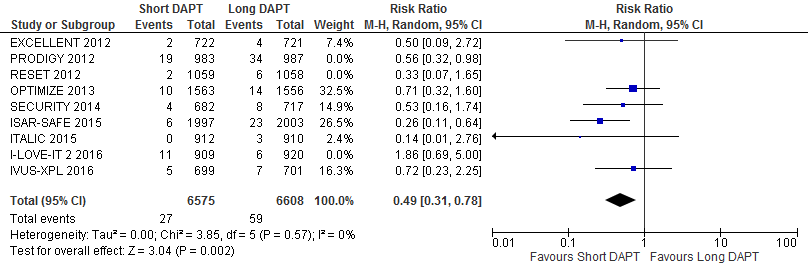


**2.2 high numbers of patients with ACS (≥50%)**

All-cause mortality

**
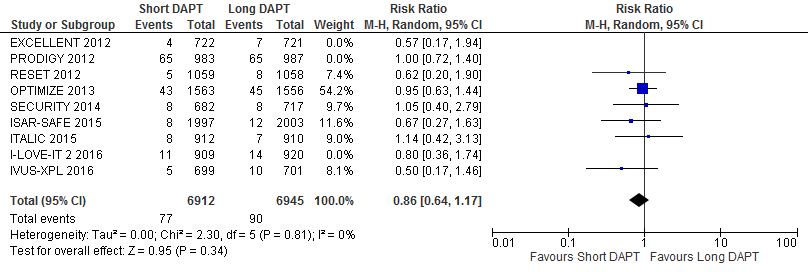
**

Myocardial infarction


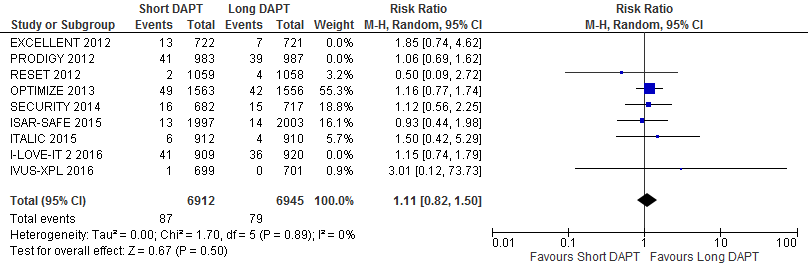


Stent Thrombosis


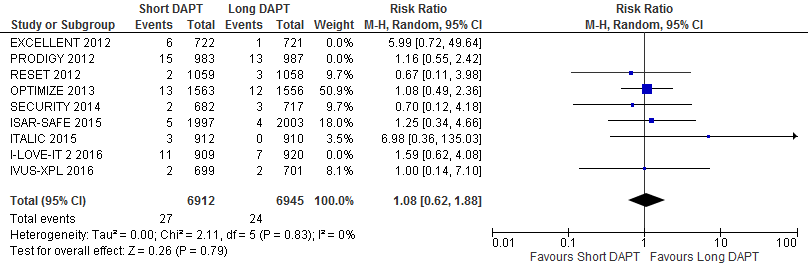


Stroke


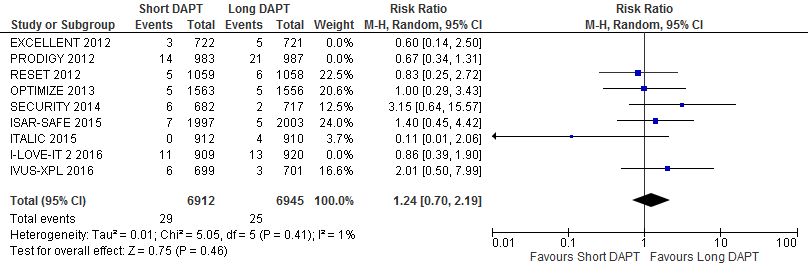


Major bleeding


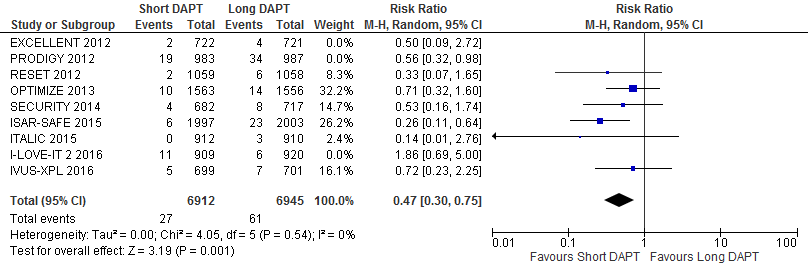


**2.3 first-generation DES (≥25%)**

All-cause mortality


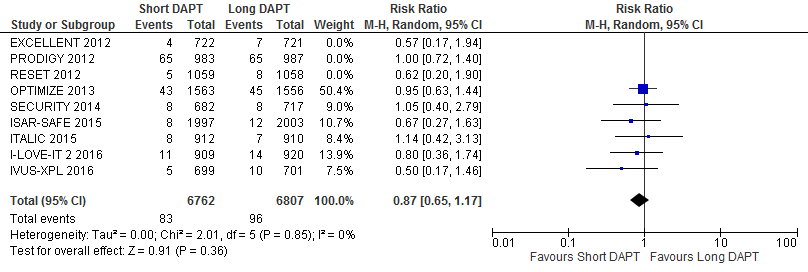


Myocardial infarction


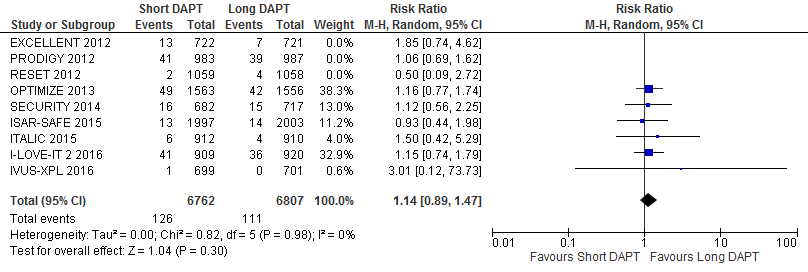


Stent Thrombosis


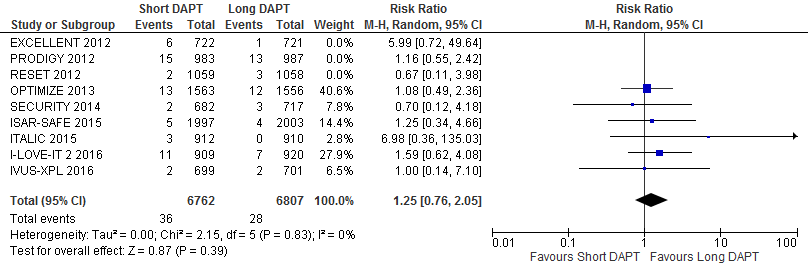


Stroke

**
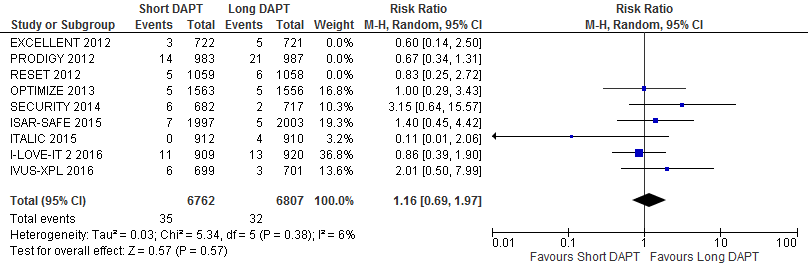
**

Major bleeding

**
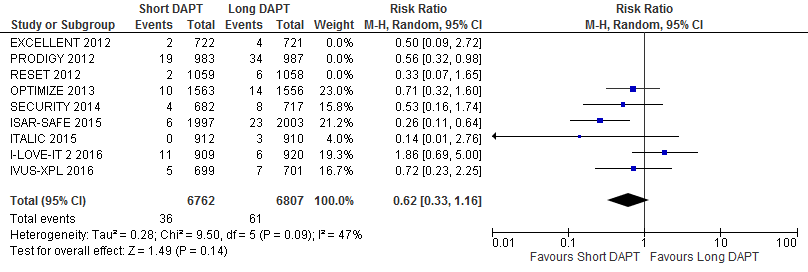
**

**2.5 high number of complex lesions (≥60%)**

All-cause mortality


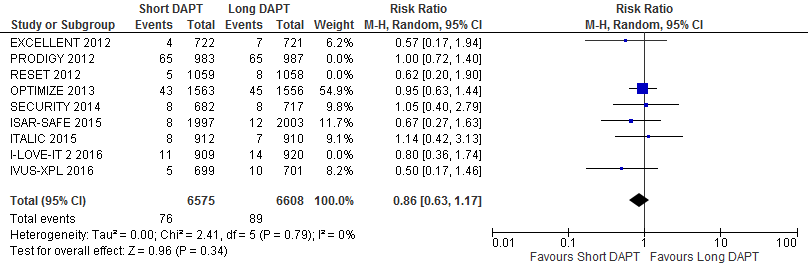


Myocardial infarction


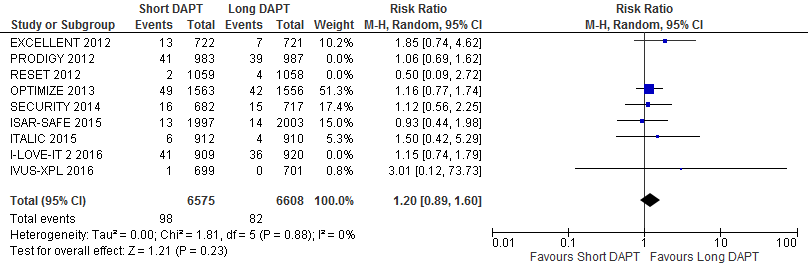


Stent Thrombosis


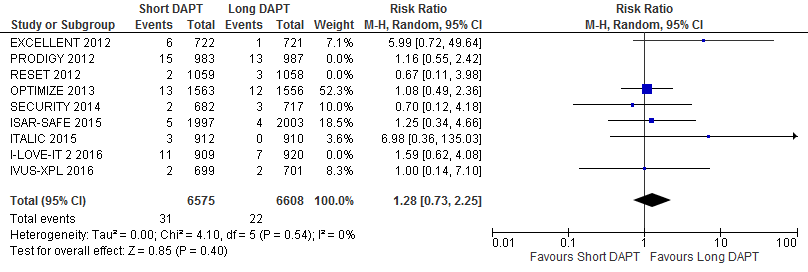


Stroke


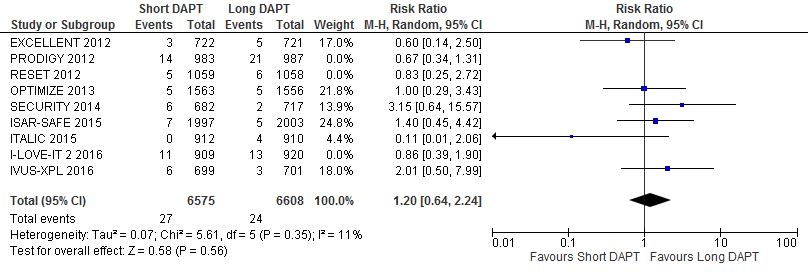


Major bleeding


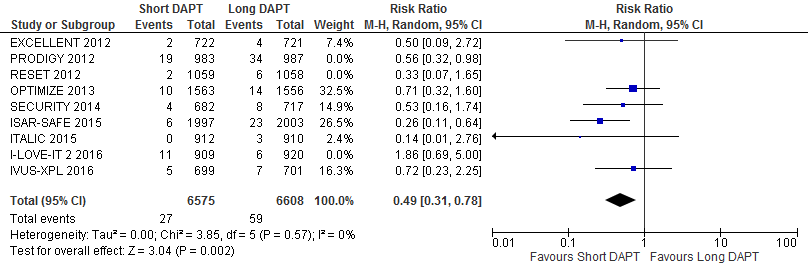

Supplement: Supplementary file 1 — Search strategy, PRISMA-flow chart, risk of bias evaluation, and additional trial characterics. Supplementary results include additional analysis to investigate the impact of studies with STEMI (≥10%), ACS (≥50%), first-generation DES (≥25%), and complex lesions (≥60%) [file 12471_2018_1104_MOESM1_ESM.docx]
